# Response generation, not response execution, influences feelings of rightness in reasoning

**DOI:** 10.1177/17470218231156712

**Published:** 2023-03-26

**Authors:** Kaiden M Stewart, Evan F Risko, Jonathan Fugelsang

**Affiliations:** Department of Psychology, University of Waterloo, Waterloo, Ontario, Canada

**Keywords:** Reasoning, decision-making, metacognition, fluency

## Abstract

It has been argued that the experience of *ease* (i.e., the ability to quickly generate an initial response) during processing influences one’s likelihood of engaging reflectively when reasoning. This is a key facet of Metacognitive Reasoning Theory (MRT) and numerous studies have found support for this claim by showing that answers that come to mind quickly, are associated with higher feelings of rightness (FORs), and less reflective processing. However, the possibility remains that the critical determinant of FORs may be the speed of executing a response and not generating a response, given the nature of the evidence for this claim. Across two experiments, we manipulated the duration of the response execution to identify whether participants’ FOR judgements are at least partially based on factors occurring after the initial mental generation of an answer. We found no evidence that FORs nor reflection are influenced by a manipulation of response execution. Broadly, the present investigation provides evidence that the relation between speed of response and FORs is likely due to the speed with which an answer is generated internally, and not the response execution phase. These findings are consistent with Metacognitive Reasoning Theory and provide further support for the suggestion that answer fluency is the critical variable in determining FORs. All data, scripts, and materials can be found at https://osf.io/f48az/

Dual-process theories of reasoning suggest that, when making decisions, individuals produce two qualitatively distinct types of response. Type I responses are quick, and based on intuitive, heuristic processes, and sometimes individuals override these responses with a slower, reflective, deliberative, Type II response (De Neys, 2006; Evans, 2003, 2007, 2008; Evans & Stanovich, 2013). Although the common conceptualisation of Type I processes as “always bad” and Type II processes as “always good” is fallacious (Evans & Stanovich, 2013; see Evans, 2007 for examples), Type II reasoning is thought to be necessary to engage in cognitive decoupling (i.e., abstracting the problem so as to reduce influence from real-world beliefs; Stanovich, 2011, 2012). Cognitive decoupling, in turn, is necessary to solve many of the abstract, representational problems studied in the reasoning domain (Evans, 2010). As such, much attention has been given to the characteristics of problems (e.g., De Neys & Glumicic, 2008; Pennycook et al., 2012), and those who are solving them (e.g., Frederick, 2005; Littrell et al., 2019; Pennycook & Rand, 2019) that are associated with an increased tendency for Type II reasoning to be engaged.

Thompson et al. (2011) argued that, in addition to characteristics of problems and problem-solvers, characteristics of the reasoning process itself can drive the ultimate decision to reflect. Specifically, the experience of *ease* (i.e., the ability to quickly generate a response) during Type I processing is held to influence one’s likelihood of engaging Type II processing. That is, Type I outputs that are generated easily will be associated with less reflection, and Type I outputs that are difficult to generate with more reflection. This framework for understanding Type II engagement is referred to as Metacognitive Reasoning Theory (MRT; Thompson and colleagues 2011). Thompson et al. (2011) provided evidence for the core claim of this account using a two-response paradigm, wherein participants were initially required to assess the logicality of a syllogism, but did so under instruction to answer quickly, with the first response that came to mind. This was done to reduce the likelihood that the initial response was influenced by Type II processes. After the initial response, participants gave a feeling-of-rightness (for) judgement about how likely they thought it was that they initially generated the correct response. Participants then responded to the syllogism again but this time were given unlimited time to try to generate the correct answer. Thompson and colleagues observed higher FORs for initial responses that were generated quickly, suggesting that responses accompanied by a feeling of ease generate greater confidence. In addition, trials with greater FORs were associated with a decreased tendency to engage Type II processes, and as such they argued that the ultimate decision to reflect is at least partially driven by the ease, or fluency, experienced when generating a Type I response.

## What is fluency?

Cognitive processes vary in terms of the effort they require to complete. It has been argued that processes requiring little effort are accompanied by a generalised feeling of ease or goodness (Alter & Oppenheimer, 2009), which has been suggested to influence various judgements about a task, including confidence (Alter et al., 2007; Hertzog et al., 2003; Simmons & Nelson, 2006). *Processing fluency* is the broad name given to this subjective feeling of ease or goodness, and considerable study has gone into determining aspects of a task that are responsible for this experience (for a review, see Alter & Oppenheimer, 2009). Similarly, much work has gone into studying the consequences of subjectively experiencing fluency. Indeed, effects of facilitating or disrupting processing on confidence or other metacognitive judgements are relatively well-documented (e.g., Castel et al., 2007; Koriat, 1993; Simmons & Nelson, 2006).

Understanding what aspects of processing influence FORs is central to better understanding the tendency to reflect. In Thompson and colleagues’ (2011) work, FORs were argued to arise from what they referred to as *answer fluency*. Answer fluency refers to the ease with which an individual can generate an answer; that is, answers that come to mind more easily are higher in answer fluency. This idea leaves out of the fluency experience, or at least the one influencing FORs, processes or events that occur after the generation of the answer (e.g., the execution of a response). In the present investigation, we examine the potential influence of the latter on FORs and reflection.

The idea that the fluency experience might be affected by different stages of processing has been explored outside the reasoning context. Reber et al. (2004) demonstrated in a word-identification task that both figure-ground contrast and font influence subjective judgements about fluency, despite hindering the perception of stimuli at different stages of processing. Although low figure-ground contrast hindered only the detection of stimuli and not their identification, and disfluent font hindered only the identification of stimuli and not their detection, both independently reduced subjective fluency judgements. This finding provided evidence that (1) individuals are sensitive to the experience of ease at multiple stages of processing and (2) that subjective judgements about ease are influenced by these experiences at different stages of processing.

The idea that post-answer processes/events might influence the experience of ease or difficulty also draws support from extant work in other domains. For example, Susser and Mulligan (2015; also Susser et al., 2017) required participants in a memory task to respond to each word in a list by writing it down. This was done either with one’s dominant or nondominant hand. Writing words with one’s nondominant hand produced decreased judgements of learning (JOLs) for those words, despite showing no detriment in recall. This finding suggests that elements of one’s response to a stimulus can act as a cue from which to make metacognitive judgements about the processing of that stimulus. Put another way, the actual physical execution of a response factors into a subjective feeling of difficulty.

In a similar vein, Undorf and Erdfelder (2011) provided evidence that individuals use time, in and of itself, as a metacognitive cue. In their studies, participants were asked to provide JOLs for another participant. However, in one condition, the only information participants had by which to make this judgement was the duration of the yoked participant’s self-paced study of the items. Perhaps unsurprisingly, participants provided greater JOLs for short study periods, presumably inferring ease on the part of the participant to whom they were yoked. However, even when they had access to the items themselves (and thus, presumably, their difficulty), short study periods were still given greater JOLs than longer study periods. That is, participants use time *per se* as a metacognitive cue, even in the light of access to subjective difficulty information. Combining the results of Undorf and Erdfelder (2011) with those of Susser and Mulligan (2015; Susser et al., 2017), one can posit that (1) individuals infer ease or difficulty from the amount of time elapsed in a given task and (2) elapsed time includes processes up until the completion of a response. Thus, it is possible that post-answer processes/events might influence FORs by virtue of the fact that they necessarily affect the time required to complete a response.

While the view that the relation between speed and FORs is a result of answer fluency is consistent with Thompson and colleagues’ data, the nature of the evidence (i.e., a correlation between RT and FOR), of course, leaves open the possibility of post-answer processes or events influencing one’s FOR. This is because, an individual’s response time, what Thompson and colleagues used as an index of answer fluency, necessarily includes both pre- and post-answer generation (though necessarily pre-response) processes. Thus, it is feasible that what predicts FORs is not the speed that an answer comes to mind, but rather the speed with which a response is completed which would include post-answer processes (e.g., response execution). We test this idea here. Specifically, the current set of experiments differentiates between the account proposed by Thomson and colleagues (2011), that faster responses are associated with higher FORs because FORs are in part due to the ease with which individuals are able to produce a response, and an alternative account that faster responses are associated with higher FORs because of some mechanism including processes that occur after a response is generated but before it is completed. For example, it could be the case that individuals monitor their responses for speed, and therefrom infer ease or difficulty—in this case, it may reasonably not be the speed with which an answer is brought to mind, but the speed with which a response is produced that is responsible for the observed relation between RT and FOR. Notably, the latter possibility is consistent with recent work in the metamemory literature referenced above. Critically, the current investigation differentiates these two accounts.

## Experiment 1

In Experiment 1, we manipulated the duration of time preceding the *physical* response to identify whether participants’ confidence (FOR) judgements are at least partially based on factors occurring after the initial *mental* generation of an answer. We utilised the two-response paradigm introduced by Thompson and colleagues (2011), but with the important difference that the execution of participants’ responses was slowed on half of trials. If it is true that participants evaluate their outputs in terms of ease or speed at the moment an answer is generated, then any manipulation beyond that point will have no effect on FORs nor reflection. If, however, participants evaluate Type I outputs based on the total time it takes to respond, a delay of said response might produce effects on FORs and reflection similar to outputs that are difficult to generate. That is, slower responses should feel less right.

### Method

#### Participants

Forty undergraduate students from the University of Waterloo participated in exchange for partial course credit. This sample size was pre-registered and based on an *a priori* power analysis and gave us 80% power to detect a medium-sized effect equivalent to *d* = .45 with a two-tailed, paired-samples *t*-test. This effect size was based on a previous version of the experiment, but it has since been determined that the results of said experiment were due to a methodological artefact conceptually unrelated to the present investigation. As such, the prior experiment and any comparisons of the present experiment to it are not reported. However, that experiment demonstrates, at the least, what size effect a manipulation of participants’ response can be expected to have on FOR judgements. For that reason, we have used that effect size as a best guess from which to determine an appropriate sample size. We have also complemented our analyses with Bayesian analyses to provide quantification of evidence in case this sample size is too small to detect observed effects.

#### Design

There was one key independent variable of theoretical interest, which had two levels (Condition: Fast vs. Slow). Each condition had its own block, which were counterbalanced across participants. The key dependent variables of interest were FORs, Response Initiation Time (time to begin a response to the first iteration of each syllogism by moving the mouse towards one of the choices; used instead of actual response time because response time necessarily includes our manipulation on half of trials), Rethinking Time (time to respond to the second iteration of each problem), and Answer Change (whether the response to the second iteration of the problem differed from that of the first iteration).

#### Stimuli and apparatus

Stimulus presentation and response recording was controlled by MATLAB 2014b using Psychophysics Toolbox 3.0.12 (Brainard, 1997; Kleiner et al., 2007). The stimuli consisted of 48 individual syllogisms, which varied systematically in terms of their type (categorical vs. conditional), validity, and believability of their premises and conclusions. To avoid issues of having very few examples of each unique kind of syllogism, each combination of syllogism type and validity had the same logical structure (see Table 1 for an example of each of the resultant structures). Participants selected their response to each syllogism using the mouse.

**Table 1.** A list of the and an example of each.

Type	Validity	Example
Conditional	Valid	If an animal is a tiger then it eats meat. If an animal eats meat then it is a carnivore. Therefore, if an animal is a tiger then it is a carnivore.
Conditional	Invalid	If a plant is a weed then it is an oak tree. If a plant is an oak tree then it is a flower. Therefore, if a plant is a flower then it is a weed.
Categorical	Valid	No butterflies are insects. Some rabbits are insects. Therefore, some butterflies are not rabbits.
Categorical	Invalid	No puppies are cobras. Some snakes are cobras. Therefore, some snakes are not puppies.

Note that the logical structure of all like items is identical; only the substance of the syllogism changes. Also note that the premises and conclusions af the first and fourth syllogisms here are believable, the second and third unbelievable. Thus, the first two syllogisms are congruent vis-à-vis belief and validity and the second two syllogisms are incongruent.

#### Procedure

On each trial, participants were required to respond to the stimulus twice. Each time they responded, they were required to select “yes” if the provided conclusion followed logically from the premises, and “no” if the provided conclusion did not follow logically from the premises. On the first iteration, it was emphasised that they respond as fast as possible, and on the second, it was emphasised that they take as long as they need to get the correct answer. After the first iteration, but before the second iteration, participants indicated on a 7-point scale how likely they thought it was that their initial response was correct (their FOR). Before each individual response, the mouse reset to the vertical and horizontal centre of the screen. Response options were equidistant (~450 pixels) to the right and left of the horizontal centre of the screen, but centred vertically. In one block of trials, the mouse was slowed to a speed of three pixels per frame during the first, speeded response. This increased the duration of response by approximately 3 seconds, which also resulted in an overall increase in Response Time. The mouse did not deviate from vertical centre of the screen and also could not travel past the response options. This functioned to maintain fidelity of RT data, such that we know that the recorded RT involved only the time it took to make the decision plus the time it took for the mouse to travel to the chosen option.

Prior to the test trials, participants were given detailed instructions about the task, including that the mouse would be slowed on some trials. They were instructed to evaluate the conclusions as if the premises were true, regardless of whether they believed the premises to be true in the real world. All participants completed two practice trials, on one of which the mouse was slowed during the first response.

### Results

All data analysis and visualisation was completed in the R programming language (R Core Team, 2017) with the assistance of add-on packages (see: apa (Gromer, 2017), BayesFactor (Morey & Rouder, 2018), dplyr (Wickham et al., 2018), ez (Lawrence, 2016), lsr (Navarro, 2015), outliers (Komsta, 2011), plyr (Wickham, 2011), stats (R Core Team, 2017)). All analyses were carried out with an α of .05, and all *t*-tests were two-tailed. Bayesian analyses were carried out using a default prior of √2/2 (Morey & Rouder, 2018). Outliers were addressed at the individual and at the group level for all analyses involving response times by removing any data points with a *Z*-score > 3. This resulted in the pre-analysis removal of 51 trials (2.65%) across all participants, including those who were removed from each individual analysis. Analysis of only the fully retainable data (i.e., those who were never an outlier) can be found in the supplemental materials, but those results are universally consistent with the ones reported here.

#### Pre-registered analyses.^1^

We first analysed our data to examine the degree to which they are consistent with the key facets of MRT. To wit, within-subjects correlations were computed between Response Initiation and FOR. We decided to correlate FOR and Response Initiation, and not Response Time, because slowed trials (i.e., half of trials) have had an approximately 3-second delay added to every trial, and this delay may not be universally consistent between trials. This makes any correlational analysis using these times difficult to interpret. The average within-subjects correlation between Response Initiation and FOR was negative and significantly different from zero (−.17), *t*(39) = 4.06, *p* < .001, *d* = 0.64, 95% CI = [−.25, –.08], BF_10_ = 114.16. Similarly, the average within-subjects correlation between FOR and Rethinking Time was also negative and significantly different from zero (–.44), *t*(39) = 15.20, *p* < .001, *d* = 2.40, 95% CI = [−.50, –.38], BF_10_ = 9.48 × 10^14^. Trials on which participants changed their answer (*M* = 3.86, *SD* = 0.83) had a lower mean FOR than did trials on which participants did not change their answer (*M* = 5.35, *SD* = 0.82), *t*(39) = 12.69, *p* < .001, *d* = 2.01, 95% CI = [−1.72, –1.25], BF_10_ = 3.21 × 10^12^. Trials on which answers changed (*M* = 20.06 s, *SD* = 9.75 s) also had a greater mean Rethinking Time than did trials where answers did not change (*M* = 11.37 s, *SD* = 7.36 s), *t*(39) = 8.25, *p* < .001, *d* = 1.30, 95% CI = [6.56, 10.83], BF_10_ = 2.45 × 10^7^. In summary, these findings are all consistent with Thompson and colleagues’ (2011), and as such any conclusions derived here from are likely generalizeable.

**Figure 1.**
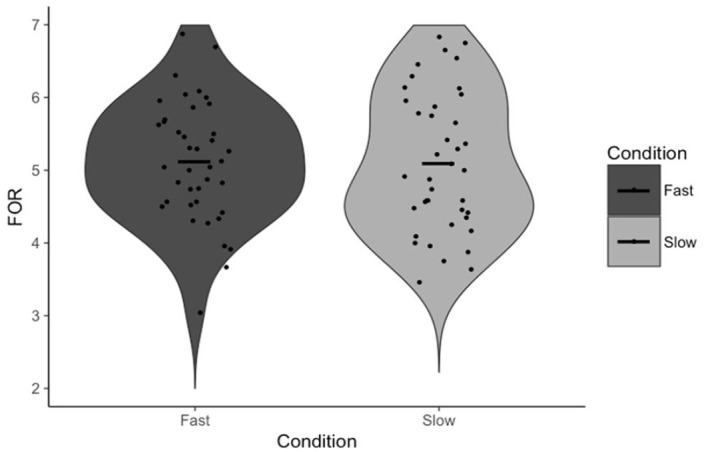
FOR as a function of condition. Points indicate individual participant means. Horizontal lines indicate the group mean.

**Figure 2.**
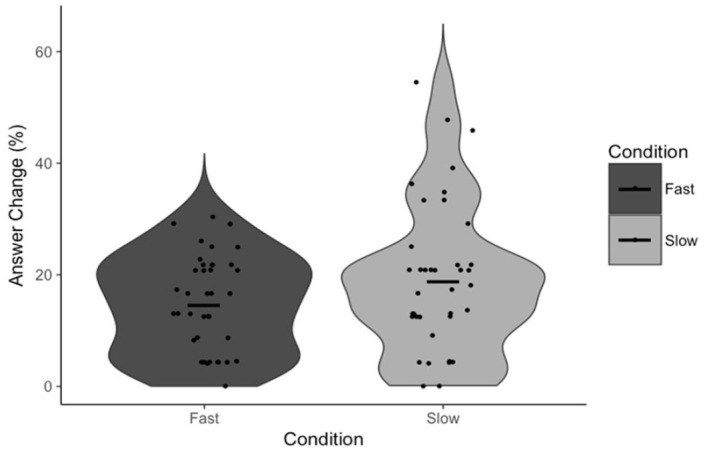
Answer Change as a function of condition. Points indicate individual participant means. Horizontal lines indicate the group mean.

**Figure 3.**
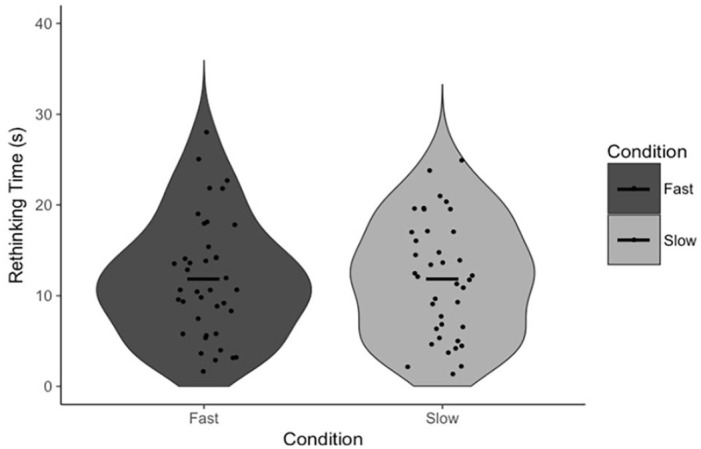
Rethinking Time as a function of condition. Points indicate individual participant means. Horizontal lines indicate the group mean.

To test our specific hypotheses, paired-samples *t*-tests were conducted to test the effect of the response execution manipulation on FOR and, independently, our measures of reflection (Answer Change and Rethinking Time). There was no statistically significant difference in FOR between fast (*M* = 5.12, *SD* = 0.82) and slow (*M* = 5.10, *SD* = 0.96) trials, *t*(39) = 0.19, *p* = .853, *d* = 0.03, 95% CI = [−0.20, 0.24], BF_01_ = 5.77 (Figure 1). There was also no statistically significant difference in Answer Change between fast (*M* = 14.59%, *SD* = 8.73%) and slow (*M* = 18.86%, *SD* = 13.67%) trials, *t*(39) = 1.85, *p* = .072, *d* = 0.29, 95% CI = [−8.95, 0.40], BF_01_ = 1.25 (Figure 2). Note that, in the latter case, the Bayesian analysis suggests no substantial evidence in favour of either the null nor alternative hypothesis, though we have marginally more evidence for the former. There was no statistically significant difference in Rethinking Time between fast (*M* = 11.85 s, *SD* = 6.58 s) and slow (*M* = 11.93 s, *SD* = 6.43 s) trials, *t*(38) = 0.09, *p* = .931, *d* = 0.01, 95% CI = [−1.93, 1.77], BF_01_ = 5.77 (Figure 3). The latter analysis was, in error, not pre-registered.

#### Exploratory analyses

Given our response execution manipulation was blocked and counterbalanced, we analysed whether our measures of reflection differed across blocks. We found that participants’ Rethinking Time was lower in Block 2 (*M* = 10.04 s, *SD* = 6.06 s) than in Block 1 (*M* = 13.74 s, *SD* = 6.39 s), *t*(38) = 5.35, *p* < .001, *d* = 0.86, 95% CI = [2.30, 5.10], BF_10_ = 4,225. In addition, participants were less likely to change their answer in Block 2 (*M* = 13.09%, *SD* = 11.55%) than in Block 1 (*M* = 20.36%, *SD* = 10.58%), *t*(39) = 3.44, *p* = .001, *d* = 0.54, 95% CI = [3.00, 11.54], BF_10_ = 15.87. Correspondingly, mean FORs were higher in Block 2 (*M* = 5.24, *SD* = 0.97) than in Block 1 (*M* = 4.98, *SD* = 0.79), *t*(39) = 2.52, *p* = .016, *d* = 0.40, 95% CI = [−0.46, –0.05], BF_10_ = 2.74. There were no effects of the order in which the blocks were seen on FORs (*M*_FS_ = 5.09, *SD*_FS_ = 0.81; *M*_SF_ = 5.12, *SD*_SF_ = 0.85), *t*(38) = 0.09, *p* = .928, *d* = 0.03, 95% CI = [−0.51, 0.56], BF_01_ = 3.27, Answer Change (*M*_FS_ = 15.74%, *SD*_FS_ = 9.14%; *M*_SF_ = 17.82%, *SD*_SF_ = 8.56%), *t*(38) = 0.74, *p* = .461, *d* = 0.24, 95% CI = [−3.58, 7.75], BF_01_ = 2.60, nor Rethinking Time, (*M*_FS_ = 10.95 s, *SD*_FS_ = 5.81 s, *M*_SF_ = 14.34 s, *SD*_SF_ = 8.59 s), *t*(38) = 1.46, *p* = .151, *d* = 0.46, 95% CI = [−1.30, 8.08], BF_01_ = 1.40.

Participants were more accurate when the mouse was not slowed (*M* = 62.7%, *SD* = 12.5%) than when it was slowed (*M* = 56.7%, *SD* = 17.0%) during the initial response, *t*(39) = 2.34, *p* = .024, *d* = 0.37, 95% CI = [0.83, 11.22], BF_01_ = 1.93, but note that the Bayesian analysis is inconclusive. There was no difference in accuracy for the second response between fast (*M* = 63.1%, *SD* = 13.0%) and slow (*M* = 60.76%, *SD* = 16.34%) trials, *t*(39) = 1.10, *p* = .278, *d* = 0.17, 95% CI = [−1.95, 6.61], BF_01_ = 3.34. In addition, we tested whether there were differences in the initiation of response as a function of Condition. There was no statistically significant difference in Response Initiation between fast (*M* = 7.10 s, *SD* = 1.85 s) and slow (*M* = 7.21 s, *SD* = 2.21 s) trials, *t*(37) = 0.45, *p* = .655, *d* = 0.07, 95% CI of the difference [−0.62, 0.39], BF_01_ = 5.21. Taken together, these analyses suggest there was probably no substantial difference in the response generation process depending on whether the mouse was slowed or regular speed.

As a robustness check, we analysed our data in a similar manner to past investigations regarding syllogistic reasoning. We were specifically interested in the effects of all the characteristics of our syllogisms (Type, Validity, Premise Believability, and Conclusion Believability) on FORs and the effects of some of them (Premise and Conclusion Believability) on endorsement rates and whether these effects interacted with our manipulation. Due to the limited number of observations per cell when all of these characteristics are included, we decided to conduct individual analyses of variance (ANOVAs) with each characteristic and Condition as independent variables. Any three-way interactions were thus not tested, as they would likely have too few observations per cell to be reliable. Participants’ FORs were greater after a correct response than an incorrect response, *F*(1, 39) = 9.97, *p* = .003, $\eta_{g}^{2}$ = .02, BF_10_ = 4.85, and when the syllogism was valid as opposed to when it was invalid, *F*(1, 39) = 19.19, *p* < .001, $\eta_{g}^{2}$ = .03, BF_10_ = 3,159. They were also more confident for categorical as opposed to conditional syllogisms, *F*(1, 39) = 9.71, *p* = .003, $\eta_{g}^{2}$ = .02, BF_10_ = 14.52. FORs were greater when the premises of the syllogism were believable as compared to unbelievable, *F*(1, 39) = 9.33, *p* = .004, $\eta_{g}^{2}$ = .01, BF_10_ = 5.12, but not when the conclusions were believable compared to unbelievable, *F*(1, 39) = 1.37, *p* = .249, $\eta_{g}^{2}$ < .01, BF_01_ = 5.22. There was a statistically significant Premise Believability by Conclusion Believability interaction, *F*(1, 39) = 20.95, *p* < .001, $\eta_{g}^{2}$ = .02, BF_10_ = 176.44, such that the effect on FOR was largest when both the premises and conclusions were believable. None of the characteristics of the syllogisms interacted with our manipulation, all *F*s ≤ ≤ 1.36. In terms of endorsement rates (i.e., participants’ likelihood of responding “valid”), participants were more likely to endorse as valid syllogisms that had believable premises, *F*(1, 39) = 6.54, *p* = .015, $\eta_{g}^{2}$ = .02, BF_10_ = 1.29, or believable conclusions, *F*(1, 39) = 57.76, *p* < .001, $\eta_{g}^{2}$ = .19, BF_10_ = 1.73 × 10^15^, and there was a statistically significant Premise Believability by Conclusion Believability interaction, *F*(1, 39) = 18.85, *p* < .001, $\eta_{g}^{2}$ = .05, BF_10_ = 348.73, such that the effect of believability was stronger when both premise and conclusion were believable. There was no such Conclusion Believability by Validity interaction, *F*(1, 39) = 1.13, *p* = .294, $\eta_{g}^{2}$ < .01, BF_10_ = 1.23.

### Discussion

First, our data support the claims made by Thompson and colleagues (2011) that FOR is negatively associated with reflection (on the basis of the negative correlation between FOR and Rethinking Time) and that FOR itself is negatively associated with response time (on the basis of the negative correlation between Response Initiation and FOR). The response execution manipulation had no effect on participants’ FOR judgements, nor did it have any effect on either of the measures of reflection. Though there was a marginally significant effect on Answer Change, examination of Figure 2 confirms that this effect is driven by a small number of participants, outside of which there is, reasonably clearly, no effect. These results suggest that participants are not using the total duration of their response from which to infer a sense of the difficulty of the task nor the likelihood they are correct. More specifically, it appears as if Answer Fluency, as opposed to some feeling of fluency derived from the totality of a response, produces participants’ FORs. In other words, the proposed alternative explanation for these correlations is not supported by the results of Experiment 1, and as such, Experiment 1 provides indirect evidence for the specific mechanism proposed by Thompson and colleagues.

It is worth noting that in Experiment 1 we observed significant block effects suggesting, over the course of the experiment, our participants were spending considerably less time on task. Participants reflected less, were quicker to respond in the second block, and had increased FORs, regardless of which order the blocks were presented. These time-on-task effects are ostensibly consistent with MRT; over time, perhaps because the task becomes more familiar, participants report increased FORs, and these increased FORs are accompanied by a decrease in reflection. That there was no effect of order on any of our key DVs suggests that the effects of experiment fatigue likely average out across counterbalances, but it is nevertheless possible that participants’ altered psychological state, as evidenced by a change in performance from the first half of the experiment to the second, affected their experience of ease in the task so as to suppress a real and meaningful effect of our manipulation.

## Experiment 2

In Experiment 2, we altered our experimental procedure so that the response execution manipulation was randomly intermixed, as opposed to blocked and counterbalanced. As such, participants did not know whether the mouse would be slowed on each individual trial until they began their initial response by moving the mouse from the centre of the screen.

### Method

#### Participants

Forty undergraduate students from the University of Waterloo participated in exchange for partial course credit. The sample size was arrived at in an identical fashion as in Experiment 1 and allowed us 80% power to detect an effect of *d* = .45 with a two-tailed, paired-samples t-test.

#### Design

The experimental design and materials were identical to that of Experiment 1.

#### Procedure

The procedure was exactly the same as in Experiment 1, with the exception that the response execution manipulation (i.e., the slowing of the mouse) was randomly intermixed within each subject at the trial level, rather than blocked and counterbalanced as in Experiment 1.

### Results

#### Pre-registered analyses.^2^

Forty-one trials (2.14%) were removed as outliers by the same procedure as in Experiment 1. Once again, our data are consistent with the key facets of MRT. The average within-subjects correlation between Response Initiation and FOR was negative and significantly different from zero (–.17), *t*(39) = 4.63, *p* < .001, *d* = 0.73, 95% CI = [−.25, –.10], BF_10_ = 555.57. Similarly, the average within-subjects correlation between FOR and Rethinking Time was also negative and significantly different from zero (–.42), *t*(39) = 13.34, *p* < .001, *d* = 2.11, 95% CI = [−.48, –.36], BF_10_ = 1.50 × 10^13^. Trials on which participants changed their answer (*M* = 3.36, *SD* = 0.97) had a lower mean FOR than did trials on which participants did not change their answer (*M* = 4.64, *SD* = 0.85), *t*(38) = 9.69, *p* < .001, *d* = 1.55, 95% CI = [−1.55, –1.01], BF_10_ = 1.11 × 10^9^. Trials on which answers changed (*M* = 18.80 s, *SD* = 7.81 s) also had a greater mean Rethinking Time than did trials where answers did not change (*M* = 12.21 s, *SD* = 6.00 s), *t*(38) = 10.61, *p* < .001, *d* = 1.70, 95% CI = [5.33, 7.85], BF_10_ = 1.25 × 10^10^.

**Figure 4.**
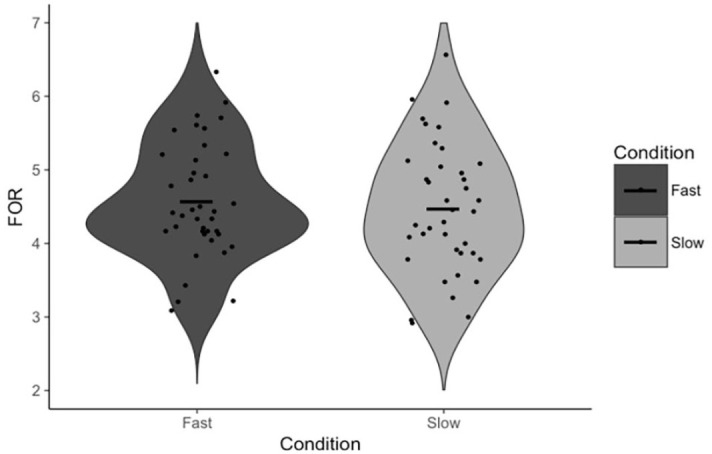
FOR as a function of condition. Points indicate individual participant means. Horizontal lines indicate the group mean.

**Figure 5.**
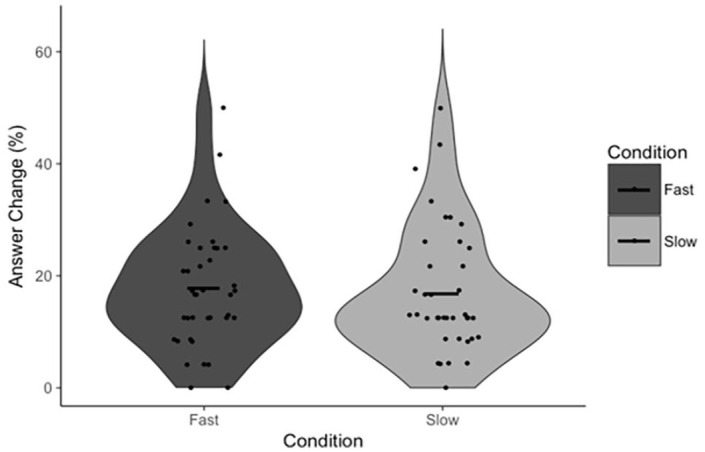
Answer Change as a function of condition. Points indicate individual participant means. Horizontal lines indicate the group mean.

**Figure 6.**
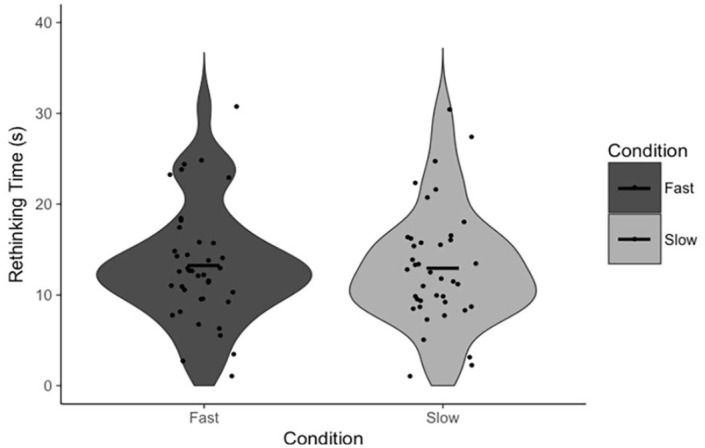
Rethinking Time as a function of condition. Points indicate individual participant means. Horizontal lines indicate the group mean.

Paired-samples *t*-tests were once again conducted to test the effect of the response execution manipulation on FOR and our measures of reflection. There was no statistically significant difference in FOR between fast (*M* = 4.57, *SD* = 0.78) and slow (*M* = 4.48, *SD* = 0.89) trials, *t*(38) = 1.24, *p* = .222, *d* = 0.20, 95% CI = [−0.06, 0.25], BF_01_ = 2.85 (Figure 4). There was also no statistically significant difference in Answer Change between fast (*M* = 17.78%, *SD* = 10.74%) and slow (*M* = 16.85%, *SD* = 11.61%) trials, *t*(38) = 0.50, *p* = .621, *d* = 0.08, 95% CI = [−2.83, 4.67], BF_01_ = 5.16 (Figure 5), nor in Rethinking Time between fast (*M* = 13.28 s, *SD* = 6.40 s) and slow (*M* = 13.01 s, *SD* = 6.40 s) trials, *t*(39) = 0.83, *p* = .411, *d* = 0.13, 95% CI = [−0.38, 0.90], BF_01_ = 4.24 (Figure 6). The comparison of Rethinking Time was not pre-registered.

#### Exploratory analyses

To mirror the block analysis from Experiment 1, we analysed differences in FOR, Rethinking Time, and Answer Change between the first half of trials and the second half of trials. There was no statistically significant difference in FOR between the first half of trials (*M* = 4.52, *SD* = 0.77) and the second half (*M* = 4.53, *SD* = 0.93), *t*(38) = 0.07, *p* = .945, *d* = 0.01, 95% CI = [−0.20, 0.19], BF_01_ = 5.78. There was also no statistically significant difference in Answer Change between the first half of trials (*M* = 18.79%, *SD* = 11.79%) and the second half (*M* = 15.68%, *SD* = 12.00%), *t*(38) = 1.36, *p* = .182, *d* = 0.22, 95% CI = [−1.52, 7.74], BF_01_ = 2.48, but note that the Bayesian analysis provides no substantial evidence for the null or alternative hypothesis. There was, however, a statistically significant difference in Rethinking Time between the first half of trials (*M* = 14.20 s, *SD* = 7.15 s) and the second half (*M* = 12.10 s, *SD* = 6.01 s), *t*(39) = 3.46, *p* = .001, *d* = 0.55, 95% CI = [0.87, 3.32], BF_10_ = 23.56.

Participants’ accuracy was nearly identical in the Fast (*M* = 60.44%, *SD* = 13.40%) and Slow (*M* = 60.98%, *SD* = 15.31%) conditions for the first response, *t*(39) = 0.18, *p* = .857, *d* = 0.03, 95% CI = [−6.61, 5.52], BF_01_ = 5.77, and the second response (*M*_F_ = 63.07%, *SD*_F_ = 14.24%; *M*_S_ = 63.62%, *SD*_S_ = 15.17%), *t*(39) = 0.17, *p* = .864, *d* = 0.03, 95% CI = [−6.98, 5.89], BF_01_ = 5.78. There was, once again, no difference in Response Initiation Time between fast (*M* = 8.78 s, *SD* = 3.24 s) and slow (*M* = 8.61 s, *SD* = 3.10 s) trials, *t*(39) = 0.78, *p* = .438, *d* = 0.12, 95% CI = [−0.27, 0.61], BF_01_ = 4.40.

As with Experiment 1, we also analysed our data in a similar manner to past investigations of syllogistic reasoning (i.e., with each characteristic of the syllogisms being analysed in an ANOVA). Participants’ FORs were greater following a correct response than following an incorrect response, *F*(1, 39) = 19.94, *p* < .001, $\eta_{g}^{2}$ = .03, BF_10_ = 385.46, and when the syllogism was valid as opposed to when it was invalid, *F*(1, 39) = 32.01, *p* < .001, $\eta_{g}^{2}$ = .04, BF_10_ = 158,527. They were also more confident for categorical as opposed to conditional syllogisms, *F*(1, 39) = 25.23, *p* < .001, $\eta_{g}^{2}$ = .03, BF_10_ = 18,126, when the premises of the syllogism were believable, *F*(1, 39) = 25.23, *p* < .001, $\eta_{g}^{2}$ = .03, BF_10_ = 1.27 × 10^8^, and when the conclusions were believable, *F*(1, 39) = 17.22, *p* < .001, $\eta_{g}^{2}$ = .02, BF_10_ = 491.75. This time, there was no statistically significant Premise Believability by Conclusion Believability interaction, *F*(1, 39) = 2.64, *p* = .112, $\eta_{g}^{2}$ < .01, BF_01_ = 4.48. None of the characteristics of the syllogisms interacted with our manipulation, all *F*s ≤ 1.62. In Experiment 2, participants were no more likely to endorse as valid syllogisms that had believable premises, *F*(1, 39) = 0.56, *p* = .459, $\eta_{g}^{2}$ < .01, BF_01_ = 21.02, but were more likely to endorse as valid those that had believable conclusions, *F*(1, 39) = 72.54, *p* < .001, $\eta_{g}^{2}$ = .35, BF_10_ = 7.02 × 10^25^, and there was no statistically significant Premise Believability by Conclusion Believability interaction, *F*(1, 39) = 1.53, *p* = .223, $\eta_{g}^{2}$ < .01, BF_01_ = 9.47. There was no Conclusion Believability by Validity interaction, *F*(1, 39) = 1.68, *p* = .203, $\eta_{g}^{2}$ < .01, BF_10_ = 1.53.

Finally, we compared all of our dependent variables of interest in Experiment 1 to Experiment 2. Although the lack of an effect of our manipulation was clearly constant across experiments, it is possible that participants’ FORs, reflection, or just performance in general changes when the manipulation is intermixed as opposed to blocked, and it is possible that these changes are informative in and of themselves. There was no difference in accuracy for the first response between Experiment 1 (*M* = 59.74%, *SD* = 12.45%) and Experiment 2 (*M* = 60.73%, *SD* = 10.82%), *t*(78) = 0.38, *p* = .705, *d* = 0.08, 95% CI = [−6.18, 4.20], BF_01_ = 4.04, nor was there a difference in accuracy for the second response between Experiment 1 (*M* = 61.94%, *SD* = 13.15%) and Experiment 2 (*M* = 63.38%, *SD* = 10.72%), *t*(78) = 0.53, *p* = .595, *d* = 0.12, 95% CI = [−6.78, 3.91], BF_01_ = 3.80. There was, however, a statistically significant difference in Response Initiation Time between Experiment 1 (*M* = 7.16 s, *SD* = 1.89 s) and Experiment 2 (*M* = 8.70 s, *SD* = 3.10 s), *t*(76) = 2.64, *p* = .010, *d* = 0.60, 95% CI = [−2.71, –0.38], BF_10_ = 4.49. There was also a statistically significant difference in FOR between Experiment 1 (*M* = 5.11, *SD* = 0.82) and Experiment 2 (*M* = 4.52, *SD* = 0.80), *t*(77) = 3.19, *p* = .002, *d* = 0.72, 95% CI = [0.22, 0.95], BF_10_ = 16.55. There was, however, no statistically significant difference in Answer Change between Experiment 1 (*M* = 16.78%, *SD* = 8.81%) and Experiment 2 (*M* = 17.29%, *SD* = 9.55%), *t*(77) = 0.25, *p* = .805, *d* = 0.06, 95% CI = [−4.63, 3.60], BF_01_ = 4.17, nor in Rethinking Time (*M_1_* = 11.91 s, *SD_1_* = 5.85 s; *M_2_* = 13.15 s, *SD_2_* = 6.32 s), *t*(77) = 0.91, *p* = .367, *d* = 0.20, 95% CI = [−3.97, 1.49], BF_01_ = 3.00.

### Discussion

In Experiment 2, we replicated the critical patterns of data from Experiment 1. Once again, the response execution manipulation had no effect on participants’ FOR judgements, nor did it have any effect on either of the measures of reflection. This time, there was a clearer non-effect of our manipulation on Answer Change. Our results also again replicate the key predictions made by MRT, specifically that speed of first response is negatively correlated with FORs, that FORs are negatively correlated with Rethinking Time, and that changed answers are associated with decreased FORs. In addition to this, our data from both Experiments 1 and 2 generally fall in line with extant observations in the syllogistic reasoning literature. The results of Experiments 1 and 2 suggest that participants, in a two-response task, do not infer FORs from the duration with which they are able to complete a response, and as such, we have evidence that the mechanism proposed by Thompson and colleagues is indeed responsible for the relations between RT, FOR, and reflection. Importantly, the results of Experiment 2 also provide a replication of the results from Experiment 1, one benefit of which is to rule out the possibility that the results in Experiment 1 were due to the blocked nature of the response execution manipulation.

## General discussion

Across two experiments, we manipulated the speed of responding in a two-response reasoning task. The purpose of this manipulation was to identify whether processes occurring after the *mental* generation of an answer (but before the *physical* completion of the response) factor into metacognitive feelings about a response, and specifically FORs. The speed manipulation impacted only processes occurring post-response generation and very likely did not cause differences before the generation of the response, as evidenced by a lack of conclusive effect on accuracy or response initiation speed. In addition, the stimulus disappeared from the screen once each participant began responding to not change the processes involved in responding for slow compared to fast trials.

It has been previously demonstrated (Thompson et al., 2011) that answers that come to mind more easily are associated with greater FORs and are subsequently associated with a decreased tendency to reflect. Critically, the operationalization of ease of responding was the speed with which a response could be completed. From the Dual-Process perspective, easier Type I outputs are less likely to be reflected upon; more difficult Type I outputs are more likely to be reflected upon. The response execution manipulation employed here differentiated two possible accounts: 1) that the relation between speed and FORs is due to the ease with which an answer comes to mind, and 2) that this relation is based on the totality of each response, including aspects of said response occurring after the point when an answer comes to mind. In the present investigation, we found no evidence that FORs nor reflection are influenced by a manipulation of response execution. In addition, our data are consistent with the key predictions of MRT; FORs were negatively correlated with both Response Time (as approximated here by Response Initiation Time) and Rethinking Time, and participants were less likely to change answers when higher FORs were given. As such, our results are very likely generalizeable to initial investigations of MRT (i.e., Thompson et al., 2011) and also other syllogistic reasoning work. That is, it is unlikely something unusual is happening in the present investigation that renders these results unique or ungeneralizeable. The results of the present investigation suggest that metacognitions in reasoning are not driven by inferences made about the length of time it takes to complete a response to a problem. That is, the mechanism we hypothesised as a possible alternative explanation of the relations between RT, FOR, and reflection, is unsupported by the evidence. More broadly, the present investigation provides support the claim that the relation between speed of response and FORs is likely due to the speed with which an answer is generated.

### Implications for fluency research

The evidence presented here is based on only one of many possible post-response production manipulations. The manipulation used herein impacted only the length of time with which a response could be completed. The observed lack of effect of the chosen manipulation precludes only a mechanism wherein time elapsed during a response is the critical factor. Perhaps a manipulation that makes responses more difficult (and is longer as a by-product of this difficulty) reduces FORs. In this vein, the results of the present investigation should not be taken as contradictory to those presented by Susser and Mulligan (2015; and Susser et al., 2017), who observed decreased JOLs for words written with one’s nondominant hand. Their handedness manipulation slowed responding to the presented stimuli but was also inherently more difficult. In addition, it is of course possible that the aspects of processing that influence FORs and JOLs are different.

One possible difference between the work presented here and past work is the obvious irrelevance of the response execution manipulation. As noted by Alter and Oppenheimer (2009; and Oppenheimer, 2004), participants will in some contexts spontaneously discount manipulations they perceive as having no relevance to the task at hand. It is therefore possible that participants in the present experiments simply discounted the response execution manipulation as a source of fluency, ignoring any potential effects of said manipulation when responding with their FORs. The manipulation used in the present investigation was designed to affect only the time it took to complete a response, and a necessary tradeoff in using a manipulation so one-dimensional is a corresponding increase in its obviousness. It is also crucial to the herein hypothesised mechanism (that participants will use an objective measure like time to circumvent the need for any online metacognitive monitoring) that the difference in time per condition be clearly noticeable to participants. In addition, the characteristics of fluency manipulations that make them susceptible to spontaneous discounting are ill-defined. As such, it is possible that the response execution manipulation was simply discounted by participants, but this limitation is seemingly necessary for an investigation like the one here.

### Implications for dual-process research

It has been argued that the driving force behind the relation between speed and FORs is that quick responses are experienced as easier. This could be true in one of two ways. One possibility is that individuals infer ease from the speed with which they can bring an answer to mind. That is, individuals might, upon the production of a response, evaluate the amount of time it took to produce said response, and infer ease if the response was produced quickly. Alternatively, individuals might simply have access to the ease or difficulty of producing a response. In this case, the relation between time to response and FORs would simply be a by-product of the relation between speed and ease. That is, it would necessarily be true that easier responses are produced more quickly. It is worth noting that the present investigation cannot differentiate between these competing explanations, as our manipulation occurred necessarily after the point where an answer had come to mind. Rather, the above accounts can both equally explain the results herein and those of Thompson and colleagues (2011).

Irrespective of the exact monitoring mechanism, this facet of dual-process decision-making has important implications. In MRT, the experience of difficulty is an important determinant of whether individuals will choose to engage their more reflective, Type II processes. In studies containing tasks like the ones contained herein, it is not always straightforward to observe reflection captured in task performance, hence why we have focused on two variables we argue capture reflection more directly. Some investigations use tasks that are argued to directly capture reflection (wherein performance is argued to be a direct measure of reflection). Either way, reflection is held as consequential and beneficial to decision quality. As such, manipulations that induce feelings of difficulty have the potential to increase reflectiveness, and downstream, improve the quality of decisions. The present investigation provides evidence suggesting that successful manipulations of the fluency experience in reasoning will likely have to operate in the time before the generation of a Type I output, as this is likely the point when a feeling of ease or difficulty arises.

Through exploratory analyses, we found two novel patterns of data that are interesting vis-à-vis MRT. First, when the response execution manipulation was blocked, the second block was associated with increased FORs and decreased reflection, consistent with the predictions of MRT. However, when the manipulation was intermixed, there was no difference in FORs between the first half of trials and the second, but there was decreased reflection for at least one of our measures of reflection (Rethinking Time; Bayesian analyses were unable to conclusively rule out an effect on Answer Change). Second, (arguably) by virtue of intermixing the response execution manipulation in Experiment 2, FORs decreased without any effect on either measure of reflection. That is, participants felt less right on average, but without a commensurate increase in reflection (though the means were higher in the intermixed condition). These results provide evidence that the nature of metacognitive experience can potentially change both over time and with the nature of the manipulation (i.e., whether it is mixed or blocked). In addition, the predicted relations between FORs and reflection are, in the data presented here, inconsistent when looking at aggregate changes; an aggregate change in FOR is not necessarily predictive of an opposite aggregate change in reflection. Put another way, while maintaining the individual correlations between FORs and reflection, MRT seems not to capture aggregate changes. Although Thompson and colleagues primarily conceptualise MRT within an individual’s metacognitive experiences (i.e., trials on which that individual experiences ease will be met with relatively little reflection for that individual), it is perhaps more accurate to conceptualise MRT at only the individual level. This is consistent with the view that the crucial feature of the experience of ease is that it changes relative to past experience (Hansen & Wänke, 2013; Wänke & Hansen, 2015). That is, fluency is a relative more so than an absolute phenomenon; ease relative to expectations or recent past states influences metacognitive judgements. In this case, FORs are likely a product of experienced ease relative to previously experienced ease, and thus should probably be held to be predictive of reflection at the trial level, and not be expected, when subject to aggregate changes, to produce aggregate changes in reflection. This insight might have important implications for attempts to use introduced disfluencies as a means to increase reflection.

## Conclusion

The present investigation represents a first, basic step towards ruling out alternative mechanistic explanations behind the operation of metacognitive processes when reasoning. Specifically, we provide evidence against an alternative account suggesting time *per se* is a cue by which individuals infer FOR when reasoning. Future research should further investigate the key drivers of metacognitive processes that ultimately shape reasoning performance to answer some of the questions left unanswered by the work presented here.

## Supplemental Material

sj-docx-1-qjp-10.1177_17470218231156712 – Supplemental material for Response generation, not response execution, influences feelings of rightness in reasoning Click here for additional data file. Supplemental material, sj-docx-1-qjp-10.1177_17470218231156712 for Response generation, not response execution, influences feelings of rightness in reasoning by Kaiden M Stewart, Evan F Risko and Jonathan Fugelsang in Quarterly Journal of Experimental Psychology

## Notes

1 Link to preregistration: https://osf.io/wxhtn

2 Link to preregistration: https://osf.io/7t2fa
