# Supplementary material for: Response generation, not response execution, influences feelings of rightness in reasoning
Source: Q J Exp Psychol (Hove). 2023 Mar 26;76(10):2379–89. doi: 10.1177/17470218231156712 (PMC10503250; doi:10.1177/17470218231156712)
Supplement: sj-docx-1-qjp-10.1177_17470218231156712 – Supplemental material for Response generation, not response execution, influences feelings of rightness in reasoning [file sj-docx-1-qjp-10.1177_17470218231156712.docx]

Supplementary Material for:

Response Generation, not Response Execution, Influences Feelings of Rightness in Reasoning

Kaiden M. Stewart^1^

Evan F. Risko^1^

Jonathan Fugelsang^1^

^1^ Department of Psychology, University of Waterloo

Speed of Response does not Affect Feelings of Rightness in Reasoning: Supplement

As a result of our approach to removing outliers, analysis of each dependent variable in the manuscript are based on different numbers of participants. For completeness, and to avoid any possible spurious conclusions, we report here in the supplement the same tests of our hypothesis on the largest fully retainable data. That is, only those retained in all analyses are included here.

**Experiment 1**

There was no statistically significant difference in FOR between fast (*M* = 5.13, *SD* = 0.81) and slow (*M* = 5.12, *SD* = 0.97) trials, *t*(36) = 0.01, *p* = .989, *d* = 0.002, 95% CI = [-0.23, 0.23], BF_01_ = 5.66. There was also no statistically significant difference in Answer Change between fast (*M* = 14.64%, *SD* = 8.53%) and slow (*M* = 18.89%, *SD* = 13.62%) trials, *t*(36) = 1.81, *p* = .079, *d* = 0.30, 95% CI = [-9.01, 0.51], BF_01_ = 1.29. There was no statistically significant difference in Rethinking Time between fast (*M* = 112.09 s, *SD* = 6.67 s) and slow (*M* = 11.92 s, *SD* = 6.36 s) trials, *t*(36) = 0.19, *p* = .849, *d* = 0.03, 95% CI = [-1.62, 1.96], BF_01_ = 5.56.

The average within-subjects correlation between Response Initiation and FOR was negative and significantly different from zero (-.17), *t*(36) = 4.02, *p* < .001, *d* = 0.66, 95% CI = [-.26, -.09], BF_10_ = 96.77. The average within-subjects correlation between FOR and Rethinking Time was also negative and significantly different from zero (-.44), *t*(36) = 14.75, *p* < .001, *d* = 2.42, 95% CI = [-.50, -.38], BF_10_ = 7.24 x 10^13^. Trials on which participants changed their answer (*M* = 3.88, *SD* = 0.85) had a lower mean FOR than did trials on which participants did not change their answer (*M* = 5.36, *SD* = 0.82), *t*(36) = 11.77, *p* < .001, *d* = 1.93, 95% CI [-1.74, -1.23], BF_10_ = 1.08 x 10^11^. Trials on which answers changed (*M* = 19.20 s, *SD* = 9.38 s) also had a greater mean Rethinking Time than did trials where answers did not change (*M* = 10.75 s, *SD* = 5.65 s), *t*(36) = 8.01, *p* < .001, *d* = 1.32, 95% CI [6.31, 10.58], BF_10_ = 7,113,883.

**Experiment 2**

There was no statistically significant difference in FOR between fast (*M* = 4.57, *SD* = 0.75) and slow (*M* = 4.47, *SD* = 0.86) trials, *t*(36) = 1.27, *p* = .214, *d* = 0.21, 95% CI [-0.06, 0.26], BF_01_ = 2.71. There was also no statistically significant difference in Answer Change between fast (*M* = 17.39%, *SD* = 9.16%) and slow (*M* = 16.98%, *SD* = 11.40%) trials, *t*(36) = 0.22, *p* = .828, *d* = 0.04, 95% CI [-3.39, 4.21], BF_01_ = 5.53. There was no difference in Rethinking Time between fast (*M* = 13.86 s, *SD* = 6.21 s) and slow (*M* = 13.56 s, *SD* = 6.26 s) trials, *t*(36) = 0.90, *p* = .375, *d* = 0.15, 95% CI [-0.39, 1.00] BF_01_ = 3.89.

The average within-subjects correlation between Response Initiation and FOR was negative and significantly different from zero (-.19), *t*(36) = 6.23, *p* < .001, *d* = 1.02, 95% CI = [-.26, -.13], BF_10_ = 47,215.88. The average within-subjects correlation between FOR and Rethinking Time was also negative and significantly different from zero (-.44), *t*(36) = 14.70, *p* < .001, *d* = 2.42, 95% CI = [-.50, -.38], BF_10_ = 6.50 x 10^13^. Trials on which participants changed their answer had a lower mean FOR (*M* = 3.42, *SD* = 0.95) than did trials on which participants did not change their answer (*M* = 4.73, *SD* = 0.74), *t*(36) = 9.67, *p* < .001, *d* = 1.59, 95% CI [-1.59, -1.04], BF_10_ = 2.70 x 10^7^. Trials on which answers changed (*M* = 19.57 s, *SD* = 7.20 s) also had a greater mean Rethinking Time than did trials where answers did not change (*M* = 12.60 s, *SD* = 5.86 s), *t*(36) = 11.72, *p* < .001, *d* = 1.93, 95% CI [5.76, 8.17], BF_10_ = 9.67 x 10^10^.
